# Genome-wide association with select biomarker traits in the Framingham Heart Study

**DOI:** 10.1186/1471-2350-8-S1-S11

**Published:** 2007-09-19

**Authors:** Emelia J Benjamin, Josée Dupuis, Martin G Larson, Kathryn L Lunetta, Sarah L Booth, Diddahally R Govindaraju, Sekar Kathiresan, John F Keaney, Michelle J Keyes, Jing-Ping Lin, James B Meigs, Sander J Robins, Jian Rong, Renate Schnabel, Joseph A Vita, Thomas J Wang, Peter WF Wilson, Philip A Wolf, Ramachandran S Vasan

**Affiliations:** 1 The National Heart Lung and Blood Institute's Framingham Heart Study, Framingham, MA, USA; 2 School of Medicine, Boston University, Boston, MA, USA; 3 Whitaker Cardiovascular Institute, Boston University, Boston, MA, USA; 4 School of Public Health, Boston University, Boston, MA, USA; 5 Department of Mathematics and Statistics, Boston, MA, USA; 6 Jean Mayer USDA Human Nutrition Research Center on Aging, Tufts University, Boston, MA, USA; 7 Broad Institute of Massachusetts Institute of Technology, Cambridge, MA, USA; 8 Cardiology Division, Massachusetts General Hospital, Harvard Medical School, Harvard University, Boston, MA, USA; 9 Department of Medicine, Massachusetts General Hospital, Harvard Medical School, Harvard University, Boston, MA, USA; 10 Office of Biostatistics Research, National Heart, Lung, and Blood Institute, National Institutes of Health, Bethesda, MD, USA; 11 Emory School of Medicine, Atlanta, GA, USA

## Abstract

**Background:**

Systemic biomarkers provide insights into disease pathogenesis, diagnosis, and risk stratification. Many systemic biomarker concentrations are heritable phenotypes. Genome-wide association studies (GWAS) provide mechanisms to investigate the genetic contributions to biomarker variability unconstrained by current knowledge of physiological relations.

**Methods:**

We examined the association of Affymetrix 100K GeneChip single nucleotide polymorphisms (SNPs) to 22 systemic biomarker concentrations in 4 biological domains: inflammation/oxidative stress; natriuretic peptides; liver function; and vitamins. Related members of the Framingham Offspring cohort (n = 1012; mean age 59 ± 10 years, 51% women) had both phenotype and genotype data (minimum-maximum per phenotype n = 507–1008). We used Generalized Estimating Equations (GEE), Family Based Association Tests (FBAT) and variance components linkage to relate SNPs to multivariable-adjusted biomarker residuals. Autosomal SNPs (n = 70,987) meeting the following criteria were studied: minor allele frequency ≥ 10%, call rate ≥ 80% and Hardy-Weinberg equilibrium p ≥ 0.001.

**Results:**

With **GEE**, 58 SNPs had p < 10^-6^: the top SNPs were rs2494250 (p = 1.00*10^-14^) and rs4128725 (p = 3.68*10^-12^) for monocyte chemoattractant protein-1 (MCP1), and rs2794520 (p = 2.83*10^-8^) and rs2808629 (p = 3.19*10^-8^) for C-reactive protein (CRP) averaged from 3 examinations (over about 20 years). With **FBAT**, 11 SNPs had p < 10^-6^: the top SNPs were the same for MCP1 (rs4128725, p = 3.28*10^-8^, and rs2494250, p = 3.55*10^-8^), and also included B-type natriuretic peptide (rs437021, p = 1.01*10^-6^) and Vitamin K percent undercarboxylated osteocalcin (rs2052028, p = 1.07*10^-6^). The peak **LOD **(logarithm of the odds) scores were for MCP1 (4.38, chromosome 1) and CRP (3.28, chromosome 1; previously described) concentrations; of note the 1.5 support interval included the MCP1 and CRP SNPs reported above (GEE model). Previous candidate SNP associations with circulating CRP concentrations were replicated at p < 0.05; the SNPs rs2794520 and rs2808629 are in linkage disequilibrium with previously reported SNPs. GEE, FBAT and linkage results are posted at http://www.ncbi.nlm.nih.gov/projects/gap/cgi-bin/study.cgi?id=phs000007.

**Conclusion:**

The Framingham GWAS represents a resource to describe potentially novel genetic influences on systemic biomarker variability. The newly described associations will need to be replicated in other studies.

## Background

There is intense clinical and research interest in blood and urinary biomarkers to diagnose disease, to risk stratify individuals for prognosis and potential intervention, and to provide insights into disease pathogenesis [1]. Hence, it has been proposed that biomarkers may prove useful in the goal of developing what has been referred to as "predictive, preemptive, personalized medicine" [2].

In the present analysis, we examined biomarkers involving four biological systems: inflammation, natriuretic peptides, hepatic function, and vitamins. Circulating inflammatory, natriuretic peptides [3-5], hepatic function [6,7] and vitamin [8] biomarker concentrations have been linked to increased risk of cardiovascular disease and mortality. For instance, the inflammatory marker C-reactive protein (CRP) predicts incident stroke [9], coronary heart disease [10-12], and all-cause mortality [13].

Because of their prognostic importance, there has been interest in understanding the environmental and genetic factors contributing to interindividual variability in systemic biomarker concentrations. Prior reports support the heritability of systemic biomarker concentrations reflecting inflammatory processes [14,15], natriuretic peptides activation [16], hepatic function [17,18], and vitamin metabolism [19]. The majority of prior studies examining the genetic contribution to biomarker concentrations have examined genetic linkage or variation in selected candidate genes. Although there have been some successes with both approaches [20], the specific genes contributing to variability of most circulating biomarkers are incompletely understood. We examined the relation of single nucleotide polymorphisms (SNPs) on the Affymetrix 100K chip to variation in systemic biomarker concentrations. The GWAS approach has the advantage that it is not constrained by known physiologic associations.

## Materials and methods

### Study sample

The biomarkers were assessed in the Framingham Offspring sample, which is described in the Framingham 100K Overview [21]. Briefly, the Framingham Offspring were recruited in 1971–1974 from the children (and children's spouses) of the Framingham Original Cohort [22]. The examinations and the number of participants in which the biomarkers were assessed vary by analyte, as noted in Table 1.

**Table 1:** Types of traits phenotype master trait table, exam cycle, numbers of participants in family plates with phenotype

Phenotype	Acronym	Trait N = 27*	Subject N	Offspring Exam	Adjustment* Multivariable model
**Inflammation/Oxidative Stress**					

CD40 Ligand, serum & plasma	CD40L	2	998	7	Age, sex, smoking, systolic and diastolic blood pressure, hypertension treatment, body mass index, waist circumference, Total/HDL cholesterol, triglyceride, lipid lowering medication, glucose, diabetes, aspirin, hormone replacement therapy and prevalent cardiovascular disease
C-reactive protein	CRP	5	980–1008	2, 5, 6, 7; Average: 2, 6, 7	
Intercellular adhesion molecule-1	ICAM1	1	1006	7	
Interleukin-6	IL6	1	1006		
Urinary isoprostanes/creatinine	IsoCrUrine	1	828		
Monocyte chemoattractant protein-1	MCP1	1	989		
Myeloperoxidase	MPO	1	974		
Osteoprotegerin	OPG	1	1005		
P-selectin	Pselectin	1	1007		
Tumor necrosis factor alpha	TNFA	1	753		
Tumor necrosis factor receptor-2	TNFRII	1	980		

**Natriuretic Peptides**					

N-terminal pro-atrial natriuretic peptide	ANP	1	938	6	Age, sex, BMI, SBP, HTN Rx, LDL Total/HDL, diabetes, LV mass, LA size, CVD
B-type natriuretic peptide	BNP	1	938		

**Liver Function**					

Bilirubin	Bili	1	910	2	Age, sex, BMI, HDL, HTN, diabetes, serum total protein, alcohol intake, TG, & smoking
Aspartate aminotransferase **	AST	1	904		
Alanine aminotransferase	ALT	1	904		
Alkaline phosphatase	AlkPhos	1	904		
Gamma-glutamyl transferase	GGT	1	896		

**Vitamins**					

Vitamin K plasma phylloquinone	VitKPhylloq	1	518	6/7^†^	Age, sex, SBP, DBP, BMI, waist, total/HDL, smoking, glucose, TG, diabetes, HTN Rx, lipid lowering Rx, hormone replacement Rx, asthma Rx, alcohol use, prevalent CVD
Vitamin K percentage of undercarboxylated osteocalcin	VitKPucOC	1	504		
Vitamin D plasma 25(OH)-D	VitD25OH	1	517		

*Each trait had 2 adjustment schemes web posted: age- and sex-adjusted, and multivariable-adjusted at http://www.ncbi.nlm.nih.gov/projects/gap/cgi-bin/study.cgi?id=phs000007. GEE and FBAT traits are web displayed pha001115 through pha001218; Linkage traits are located from pha002301 through pha002352. **In the present manuscript we examine the multivariable-adjusted trait, which we count as 1 trait**. Note: biomarkers were natural log transformed due to skewed distribution; **normalized deviates. ^†^Vitamin measurements straddled exams 6 & 7, covariates from same exam biomarker assayed. SBP, DBP = systolic and diastolic blood pressure, HTN Rx = hypertension treatment, BMI = body mass index, TC/HDL = total/high density lipoprotein cholesterol; TG = triglyceride, HRT = hormone replacement therapy, Rx = medication therapy, CVD = cardiovascular disease; LDL = low density lipoprotein; LV mass = left ventricular mass; LA size = left atrial size; Atrial natriuretic peptide = N-terminal pro-atrial natriuretic peptide.

### Phenotype definitions and methods

Biomarkers were measured on morning specimens after an overnight fast (typically 10 hours) between 7:30 and 9:00 am. EDTA and citrated blood collection tubes are centrifuged in a refrigerated centrifuge immediately after venipuncture. Serum blood collection tubes sit for 30 minutes after venipuncture to allow for complete clotting. Specimens are processed immediately after centrifugation. Blood samples were centrifuged and frozen at -20° (examination 2 through 4) and -80° (examinations 5 through 7). The measurement of the inflammatory markers is detailed in the inflammatory marker manual at the National Center for Biotechnology Information http://www.ncbi.nlm.nih.gov/projects/gap/cgi-bin/study.cgi?id=phs000007.

**Inflammatory biomarkers **(except CRP) were measured in duplicate with commercially available ELISA kits: R&D Systems (intercellular adhesion molecule-1, interleukin-6, monocyte chemoattractant-1 [MCP1], P-selectin, tumor necrosis factor receptor 2, high sensitivity tumor necrosis factor-α), Bender MedSystems (CD40 ligand), Oxis (myeloperoxidase), and BIOMEDICA (osteoprotegerin). High-sensitivity CRP was measured in 2002 and 2004 on examination cycle 2, 6 and 7 specimens with a Dade Behring nephelometer; the less sensitive Hemagen assay was used in 1998 for examination cycle 5 specimens. **Natriuretic peptides **were measured by Shionogi using a noncompetitive high sensitivity immunoradiometric assay [23]. **Liver function tests **were measured at examination cycle 2 by Quest Diagnostics (previously METPATH) with a variety of methods: γ-glutamyl aminotransferase was measured with spectrophotometry [7], bilirubin was measured by the colorimetric method (Dow Bilirubin Kit) [24,25]; alkaline phosphatase was measured with the kinetic method [26,27]; aspartate aminotransferase and alanine aminotransferase were measured using the kinetic method with Beckman Liquid-Stat Reagent Kit [28]. **Vitamin **K status was measured as phylloquinone concentrations with reverse phase high-performance liquid chromatography [29], and percentage of undercarboxylated osteocalcin was measured by radioimmunoassay [30,31], Vitamin D status was measured as 25(OH)D concentrations by using RIA (DiaSorin, Stillwater MN).

**Plasma **samples were used for natriuretic peptides, vitamin K phylloquinone, vitamin D, and some inflammatory markers including CD40 ligand, osteoprotegerin, P-selectin, tumor necrosis factor receptor 2, and tumor necrosis factor-α. **Serum **samples were analyzed for liver function, vitamin K, % undercarboxylated osteocalcin, and other inflammatory markers including CRP, interleukin-6, soluble intracellular adhesion molecule-1, MCP1, and myeloperoxidase concentrations. The reproducibility of the biomarkers was good; the intra-assay coefficients of variation were CD40 ligand 4.4%, interleukin-6 3.1%, intercellular adhesion molecule-1 3.1%, MCP1 4.1%, myeloperoxidase 3.0%, osteoprotegerin 3.7%, P-selectin 3.0%, tumor necrosis factor-α 8.8%, and tumor necrosis factor receptor-2 2.3%; the inter-assay coefficients of variation were brain natriuretic peptide 12.2%, n-terminal-atrial natriuretic peptide 12.7%. The Kappa statistic for 146 CRP samples run in duplicate was 0.95 [32]. Coefficients of variation for aspartate aminotransferase and alanine aminotransferase, respectively, were 10.7 and 8.3%. The coefficients of variation for low and high Vitamin K plasma phylloquinone concentrations were 15.2 and 10.9% respectively on control specimens. For low, medium and high osteocalcin concentrations used to determine Vitamin K percentage of undercarboxylated osteocalcin, the coefficients of variation were 22.3, 12.8, and 7.8%, respectively. For Vitamin D, the coefficients of variation were 8.5% and 13.2%, respectively.

### Genotyping methods

Details of the genotyping methods are available in the Framingham Heart Study 100K Overview [21]. Framingham staff extracted genomic DNA with a Qiagen Blood and Cell Culture Maxi Kit from immortalized lymphoblasts. Briefly, SNPs on the Affymetrix 100K chip were genotyped (n = 112,990 autosomal SNPs) in a sample of family members of the Original and Offspring cohorts of the Framingham Heart Study [33]. SNPs were excluded for the following reasons: minor allele frequency <10% n = 38062; call rate <80% n = 2346; Hardy-Weinberg equilibrium p-value < 0.001 n = 1595, leaving 70,987 SNPs available for analysis.

### Statistical analysis methods

We created standardized multivariable-adjusted natural log transformed biomarker residuals adjusted for the covariates listed in Table 1. The CRP average residuals were constructed as follows: (1) create age- and sex-adjusted or multivariable-adjusted residual at each of exams 2, 6 and 7; (2) take average of the residuals across exams; (3) the residual was excluded if there were not at least 2 exams for its calculation. In some instances we performed additional transformation (e.g. Winsorized models). Tobit models were used to generate residuals for the natriuretic peptides, because 2% of N-ANP levels and 30% of BNP levels were below the respective assay detection limits. Association and linkage results examining age- and sex-adjusted residuals are posted at the web site. As described in the Overview [21], we examined generalized estimating equations (GEE) and family based association testing (FBAT), assuming an additive genetic effect, to account for correlation among related individuals within nuclear families. We also used Merlin software [34] (splitting the largest families) to compute exact identity by descent linkage, with variance component analysis in SOLAR using 11,200 SNPs and short tandem repeats [35]. Traits with extreme values, as defined by 4 standard deviations away from the mean, were Winsorized at 4.0 in secondary linkage analyses to determine the sensitivity of the logarithm of the odds (LOD) score to the presence of outlier values.

## Results

Twenty-two biomarker traits (plus 4 additional CRP traits) were analyzed in 1012 Offspring participants, on log-transformed multivariable-adjusted residuals as outlined in Table 1 (minimum-maximum per phenotype n = 507–1008). The phenotypes were collected at various Framingham Offspring examinations from cycles 2 to 7. At examination cycles 2 and 7 the mean age of the participants with both phenotype and genotype data was 41 ± 10 and 59 ± 10 years, and 51.2% and 51.1% were women, respectively. For details of biomarker phenotype-genotype association refer to http://www.ncbi.nlm.nih.gov/projects/gap/cgi-bin/study.cgi?id=phs000007.

There were 58 SNPs associated with biomarker concentrations with a p < 10^-6 ^by **GEE**. The 25 most statistically significant GEE associations sorted by p-value, listed with their corresponding FBAT p-value are shown in Table 2a. MCP1 concentrations were associated with rs2494250 (p = 1*10^-14^) and rs4128725 (p = 3.68*10^-12^), both on chromosome 1, near the *FCER1A *and the *OR10J1 *genes, respectively. CRP concentrations averaged over 3 examinations (about 20 years) were associated with rs2794520 (p = 2.83*10^-8^) and rs2808629 (p = 3.19*10^-8^).

**Table 2:** Top genetic associations with biomarkers based on the lowest p value for GEE test (2a), FBAT (2b), and Linkage (2c)

2a. Top 25 associations with biomarkers based on the lowest p value of the GEE test							
**Trait**	SNP rs ID*	**Chr**	**Physical location (bp)**	**GEE P-value**	**FBAT P-value**	**IN/NEAR gene**	

Monocyte chemoattractant protein-1	rs2494250	1	156091324	1.0*10^-14^	3.5*10^-8^	*FCER1A*, *OR10J3*	
Monocyte chemoattractant protein-1	rs4128725	1	156219032	3.7*10^-12^	3.3*10^-8^	*OR10J1*	
C-reactive protein average exams 2,6,7	rs2794520	1	156491889	2.8*10^-8^	4.3*10^-5^	*CRP*	
C-reactive protein average exams 2,6,7	rs2808629	1	156489869	3.2*10^-8^	4.8*10^-5^	*CRP*	
C-reactive protein exam 6	rs2794520	1	156491889	1.3*10^-7^	3.9*10^-4^	*CRP*	
C-reactive protein exam 6	rs2808629	1	156489869	1.4*10^-7^	4.3*10^-4^	*CRP*	
Tumor necrosis factor alpha	rs7552393	1	83966572	5.1*10^-7^	0.63		
C-reactive protein exam 6	rs746961	19	35791730	7.5*10^-7^	0.03	*ZNF536*	
Bilirubin	rs17532515	4	141745043	1.0*10^-6^	9.2*10^-6^	*CLGN*, *ELMOD2*	
Alanine aminotransferase	rs1998303	9	82644535	1.1*10^-6^	0.005		
Monocyte chemoattractant protein-1	rs10489849	1	156009838	1.1*10^-6^	0.10	*IGSF4B*	
Alkaline phosphatase	rs10518765	15	52467924	1.1*10^-6^	1.7*10^-4^		
Vitamin K plasma phylloquinone	rs2387326	10	129823446	1.1*10^-6^	0.02	*PTPRE*, *MKI67*	
C-reactive protein average exams 2,6,7	rs1119582	5	125270919	1.2*10^-6^	4.2*10^-4^		
Vitamin D plasma 25(OH)-D	rs10485165	6	89169536	1.4*10^-6^	0.003		
Atrial natriuretic peptide exam 6	rs1417352	6	107005919	1.8*10^-6^	0.009		
C-reactive protein exam 2	rs583012	10	54964880	1.9*10^-6^	0.09		
Atrial natriuretic peptide exam 6	rs1486139	7	46048968	2.0*10^-6^	0.04		
Atrial natriuretic peptide exam 6	rs1486140	7	46048877	2.2*10^-6^	0.06		
Alanine aminotransferase exam 2	rs10492681	13	39705483	2.2*10^-6^	9.9*10^-5^		
Vitamin D plasma 25(OH)-D	rs10507577	13	52866092	2.6*10^-6^	0.004		
Atrial natriuretic peptide exam 6	rs1079596	11	112801829	2.6*10^-6^	0.03	*DRD2*	
Monocyte chemoattractant protein-1	rs1474747	1	155961586	2.8*10^-6^	8.7*10^-4^	*IGSF4B*	
CD40 Ligand serum	rs7778619	7	9923216	3.0*10^-6^	0.19		
CD40 Ligand serum	rs8005745	14	76473583	3.5*10^-6^	0.01		

**2b. Top 25 associations with biomarkers based on the lowest p value of the FBAT test**							

**Trait**	SNP rs ID*	**Chr**	**Physical location (bp)**	**GEE P-value**	**FBAT P-value**	**IN/NEAR gene**	

Monocyte chemoattractant protein-1	rs4128725	1	156219032	3.7*10^-12^	3.3*10^-8^	*OR10J1*	
Monocyte chemoattractant protein-1	rs2494250	1	156091324	1.0*10^-14^	3.5*10^-8^	*FCER1A*, *OR10J3*	
B-type natriuretic peptide	rs437021	1	61450291	1.5*10^-4^	1.0*10^-6^	*NFIA*	
Vitamin K % undercarboxylated osteocalcin	rs2052028	7	15789103	5.2*10^-6^	1.1*10^-6^		
CD40 Ligand plasma	rs2372184	3	65673194	0.003	2.5*10^-6^	*MAGI1*	
Urinary isoprostanes/creatinine	rs717145	20	15826091	0.003	5.0*10^-6^	*C20orf133*	
CD40 Ligand serum	rs4664604	2	153398916	0.01	8.4*10^-6^	*ARL6IP6*	
CD40 Ligand serum	rs9288125	2	153348619	0.01	9.1*10^-6^	*FMNL2*, *ARL6IP6*	
C-reactive protein exam 7	rs1363258	5	103297593	0.02	9.2*10^-6^		
Bilirubin	rs17532515	4	141745043	1.0*10^-6^	9.2*10^-6^	*CLGN*, *ELMOD2*	
Osteoprotegerin	rs496269	6	79457094	0.03	9.4*10^-6^		
C-reactive protein average 2,6,7	rs1363258	5	103297593	0.009	1.3*10^-5^		
CD40 Ligand serum	rs303939	13	71269472	0.008	1.3*10^-5^	*DACH1*	
Myeloperoxidase	rs10501981	11	100880825	1.1*10^-5^	1.4*10^-5^	*TRPC6*	
Urinary isoprostanes/creatinine	rs1461549	14	24782140	0.26	1.5*10^-5^		
Tumor necrosis factor alpha	rs2353803	7	11060282	0.03	1.5*10^-5^		
Intercellular adhesion molecule-1	rs3849944	9	27550594	5.3*10^-6^	1.5*10^-5^	*C9orf72*	
CD40 Ligand serum	rs1986743	2	153412407	0.01	1.6*10^-5^	*ARL6IP6*	
Gamma-glutamyl transferase	rs962976	12	67006894	0.002	1.6*10^-5^	*MDM1*	
C-reactive protein average 2,6,7	rs2421608	2	117013763	0.02	1.8*10^-5^		
C-reactive protein exam 2	rs642245	11	86067184	0.03	1.9*10^-5^	*ME3*	
Tumor necrosis factor receptor-2	rs248328	5	179309691	0.59	1.9*10^-5^	*TBC1D9B*, *RNF130*	
C-reactive protein exam 7	rs2390582	1	90655928	0.07	2.0*10^-5^		
Osteoprotegerin	rs9352609	6	79442188	0.04	2.0*10^-5^		
Intercellular adhesion molecule-1	rs744511	14	39166736	3.2*10^-4^	2.1*10^-5^		

**2c. Magnitude and Location of Peak LOD scores > 2.5 for regions in the Biomarker Phenotype Group**							

**Trait**	**Exam**	**Chr**	**Physical location (bp)**	**Maximum LOD**	**LOD-1.5 Interval**	**LOD+1.5 Interval**	Maximum LOD WIN*

Monocyte chemoattractant protein-1	7	1	159093573	4.96	154908901	159751221	4.38
Monocyte chemoattractant protein-1	7	10	129553148	4.03	128294406	130084334	3.23
C-reactive protein	5	1	154745847	3.53	153213133	156567571	3.28
Monocyte chemoattractant protein-1	7	17	13630703	3.33	10874193	16776778	2.54
Intercellular adhesion molecule-1	7	1	203535232	2.95	202207846	215367881	2.93
Monocyte chemoattractant protein-1	7	7	92544810	2.94	88727093	105546050	2.01
Tumor necrosis factor receptor 2	7	1	54001041	2.92	43070922	60590679	2.95
Gamma-glutamyl transferase	2	3	26424584	2.89	24621158	27418642	2.96
B-type natriuretic peptide	6	12	4140574	2.77	132045	8137669	No outliers
Gamma-glutamyl transferase	2	10	129553148	2.67	120112006	132560638	2.79
Vitamin D plasma 25(OH)-D	6/7	8	140624328	2.67	138952328	146039126	2.68
B-type natriuretic peptide	6	19	34016706	2.59	13425865	43186344	No outliers
Myeloperoxidase	7	19	11295505	2.56	3026853	16489850	2.56
Alkaline phosphatase	2	6	170538204	WIN	162441307	170788550	2.55
Osteoprotegerin	7	13	75274475	2.52	71928655	81228082	2.95

bp = base pair; Chr = chromosome; WIN = Winsorized.

dbSNP positions are from NCBI Build 35 (hg17);

LD between rs2494250 and rs4128725 (top MCP1 SNPs): D' = 0.724 and r squared = 0.196.

LD between rs2794520 and rs2808629 (top CRP SNPs): D' = 1.0 and r squared = 1.0.

*Winsorized LOD scores were run for this manuscript, and are not displayed on the web.

We estimated the amount of variability in biomarker concentrations explained by the 4 most statistically significant SNPs in the GEE model using a pseudo measure of R^2 ^based on log-likelihood estimates [36]. The two most statistically significant GEE SNPs explained about 7% and 4% of the variability in MCP1 concentrations (R^2 ^= 0.070 for rs2494250 and R^2 ^= 0.043 for rs4128725); for CRP concentrations averaged over examinations 2, 6, and 7 the two most statistically significant GEE SNPs explained 2.3% of the variability [R^2 ^= 0.023 for rs2794520 and rs2808629) [36]. We also examined the linkage disequilibrium between the most statistically significant GEE SNPs: rs2494250 and rs4128725 had a D' = 0.724 and an r^2 ^= 0.196, whereas rs2794520 and rs2808629 served as perfect proxies for each other (D' = 1; r^2 ^= 1).

With **FBAT, **11 SNPs were associated with biomarker concentrations with a p < 10^-6^. The two most statistically significant SNPs for FBAT were the same two SNPs observed with GEE: MCP1 concentrations were significantly associated with rs4128725, p = 3.28*10^-8^, and rs2494250, p = 3.55*10^-8 ^(Table 2b). In addition, B-type natriuretic peptide (rs437021, p = 1.01*10^-6^) and Vitamin K% undercarboxylated osteocalcin (rs2052028, p = 1.07*10^-6^) also were nominally statistically significantly associated.

In Table 2c we list the magnitude and location of LOD scores > 2.5 observed for the circulating biomarker traits. Because we were concerned that some of the LOD scores might be inflated by individuals with extreme marker concentrations, we reanalyzed the LOD scores on Winsorized residuals. The peak Winsorized **LOD **scores observed were for the biomarkers MCP1 (4.38, chromosome 1), and CRP (3.23, chromosome 10; 3.28, chromosome 1). Of note the 1.5 LOD support intervals for the linkage peaks on chromosome 1 included the SNPs significantly associated with MCP1 and CRP reported above (GEE model).

In an effort to potentially uncover genetic pleiotropy we display in Table 3 two ways to synthesize findings across phenotypes. We examined 3 correlated inflammatory biomarker phenotypes, interleukin-6, CRP and fibrinogen, and report SNPs that were significantly associated with all 3 phenotypes by GEE or FBAT at p < 0.01 (Table 3a). We also examined phenotypes within a specific biomarker category including CRP over multiple examinations, liver function tests and vitamin concentrations (nutrients involved in bone health [37,38]), and display in Table 3b SNPs significant by either FBAT or GEE at a p < 0.01 for all of the phenotypes in a given phenotype cluster.

**Table 3:** Combined phenotypes

Trait	SNP rs ID	Chr	Physical location (bp)	GEE P-value	FBAT P-value	IN/NEAR gene
**3a. SNPs significant for 3 correlated phenotypes at exam 7 by either GEE or FBAT at p < 0.01**						

**Interleukin-6, C-reactive protein and Fibrinogen**	rs10511884	9	31668988	5.7*10^-5^	0.0065	
	rs1887027	10	6153788	2.6*10^-4^	0.19	*IL2RA*, *RBM17*
	rs2831617	21	28481515	6.2*10^-4^	0.0027	
	rs2831620	21	28481869	6.4*10^-4^	0.0022	
	rs2831618	21	28481749	6.4*10^-4^	0.0020	
	rs2044401	9	31659518	6.6*10^-4^	0.12	
	rs1457590	3	21530978	0.0019	0.16	*ZNF659*
	rs6848323	4	113286305	0.0022	0.14	
	rs3110134	8	60260538	0.0025	0.12	
	rs2016740	4	113238018	0.0039	0.17	
	rs719006	15	59210481	0.0044	0.76	*RORA*
	rs877936	4	113238472	0.0055	0.31	
	rs1436136	4	113421130	0.0062	0.039	
	rs1436336	3	106156256	0.0067	0.0040	
	rs698270	3	137592210	0.0086	0.020	*STAG1*
	rs847428	7	16803192	0.019	0.025	
	rs2359763	3	23424931	0.024	0.0025	
	rs7969455	12	7757402	0.059	0.0015	*DPPA3*
	rs10503717	8	22634817	0.06	0.0028	
	rs4899940	14	87623621	0.11	0.0019	

**3b. Combined phenotypes within a specific biological domain**						

C-reactive protein: exams 2, 5, 6, 7	rs2808629	1	156489869	6.9*10^-5^	4.7*10^-4^	*NFIA*, *CRP*
	rs2794520	1	156491889	6.1*10^-5^	4.85*10^-4^	*FCER1A*, *CRP*
	rs6563212	13	35380415	7.3*10^-4^	0.30	*DCAMKL1*
	rs11626844	14	72413330	5.1*10^-3^	0.17	*OR10J1*, *DPF3*
	rs9319160	13	84918646	0.002	0.09	
	rs910232	1	17143820	0.002	0.01	*MAGI1*, *PADI2*
Liver function: Alkaline phosphatase; AST; ALT; GGT	rs4911146	20	32103708	0.01	8.4*10^-6^	*ARL6IP6*, *RALY*
	rs953402	3	5986639	0.01	9.1*10^-6^	*FMNL2*
Vitamin D, Vitamin K phylloquinone & Vitamin K % undercarboxylated osteocalcin	rs1376544	4	180293700	0.02	9.2*10^-6^	

Chr = chromosome;

For a given SNP, all of the phenotypes either FBAT or GEE significant if FBAT < 0.01 for particular SNP;

P-values = the geometric mean of the p-value for all traits within the biomarker cluster

In Table 4 we compared our data with previously reported phenotype-genotype associations in the published literature on systemic biomarker concentrations: bilirubin concentrations (TA repeat in ***UGT1A1***) [39,40]; CRP (***CRP***) [20,32,41-50], intercellular adhesion molecule-1 (***ICAM1***) [51-54], interleukin-6 (***IL6***) [55-62], and MCP1 (*CCL2 *= **MCP1 gene **[63,64]). Unfortunately, there were no SNPs within 60 KB of the *ICAM1 *gene on the Affymetrix 100K chip. There was no association between bilirubin concentrations and 1 SNP within 30 kb (rs741159) + 2 more SNPs within 50 kb (rs726017 and rs6752792) of a previously reported TA repeat in ***UGT1A1***. Additionally, there was no association between interleukin-6 concentrations and SNPs in the *IL6 *region despite one SNP in high LD (linkage disequilibrium; r^2 ^= 0.819) with the previously reported rs1800795 (-174G/C) SNP. Similarly, we did not observe an association between MCP1 concentrations and SNPs in the *CCL2 *region, despite one SNP with a high r^2 ^(0.956) with the SNP previously reported in the literature. For CRP concentrations, we had 2 SNPs in perfect LD with rs1205, and we observed strong evidence for replication. However, it should be noted that this association has been previously reported by Framingham investigators in unrelated participants [32]. Similarly, rs431568, which is in high LD (r^2 ^= 0.83) with 2 previously associated SNPs (rs3116653 and rs1417938), was highly associated with many of the CRP phenotypes.

**Table 4:** Comparison with the prior literature

Gene	rs number previous reports	# Affy SNPs within 60 kb	rs ID Affy SNPs	Chr	D'	r2	Distance Associated SNP	MAF	FBAT p-value	GEE p-value
**IL6**	rs1800795 = -174G/C	7	rs6461667	7	1	0.82	30098	0.36	0.09	0.66
**MCP1**	rs1024611	13	rs10491109	17	1	0.04	30762	0.15	0.048	0.13
			rs1080327		1	0.96	11878	0.25	0.78	0.35
			rs1860181		0.92	0.27	37799	0.45	0.04	0.11
			rs1860182		0.92	0.27	37649	0.45	0.046	0.11
			rs3815341		1	0.02	34637	0.05	0.001	0.50
**CRP average 2,6,7**	rs1205	37	rs1446959	1	0.56	0.12	75429	0.39	0.86	0.002
			rs1891187		0.39	0.05	53180	0.33	0.30	0.02
			rs2808629		1	1	5437	0.34	4.8*10^5^	3.2*10^-8^
			rs2794520		1	1	3417	0.34	4.3*10^-5^	2.8*10^-8^
			rs4131568		0.63	0.12	39823	0.30	0.004	0.001
	rs1417938		rs1446959		0.5	0.16	77382	0.39	0.86	0.002
			rs1891187		0.29	0.07	55133	0.33	0.30	0.02
			rs2808629		1	0.25	7390	0.34	4.8*10^-5^	3.2*10^-8^
			rs2794520		1	0.25	5370	0.34	4.3*10^-5^	2.8*10^-8^
			rs4131568		1	0.83	37870	0.30	0.004	0.001
			rs1446959		0.66	0.03	72832	0.39	0.86	0.002
	rs3093077		rs1891187		0.47	0.02	50583	0.33	0.30	0.02
			rs2808629		1	0.03	2840	0.34	4.8*10^-5^	3.2*10^-8^
			rs2794520		1	0.03	820	0.34	4.3*10^-5^	2.8*10^-8^
			rs4131568		1	0.03	42420	0.30	0.004	0.001
			rs1446959		0.51	0.17	90106	0.39	0.86	0.002
	rs3116653		rs1891187		0.31	0.08	67857	0.33	0.30	0.02
			rs2808629		1	0.25	20114	0.34	4.8*10^-5^	3.2*10^-8^
			rs2794520		1	0.25	18094	0.34	4.3*10^-5^	2.8*10^-8^
			rs4131568		1	0.83	25146	0.30	0.004	0.001

Displayed are SNPs that are either in the highest LD (r^2^) with previously reported SNPs or that have an FBAT or GEE p-value < 0.05.

For bilirubin concentrations in Framingham study unrelated participants we previously reported significant linkage to chromosome 2q telomere [39] and a significant association to a TA repeat ***UGT1A1***, there were no association between bilirubin concentrations and 1 SNP within 30 kb (rs741159) + 2 more SNPs within 50 kb (rs726017 and rs6752792). The previously reported *UGT1A1 *variant is not a SNP and therefore not in HapMap; we have no LD information and cannot assess whether the association previous reported is also present in the current sample. ***ICAM1 ***on chromosome 19 has 3 reported SNPs in literature (rs1799969, rs5491, rs5498), but there were no Affymetrix SNPs within 60 KB of the gene.

***CCL2 ***[Other associated SNPs: rs2857654, rs1024610, rs2857657 are NOT in HapMap, so no LD information was available.

***CRP ***2 SNPs are in perfect LD with rs1205. The previously reported triallelic SNP rs3091244 is not in HapMap. CRP association was previously reported in Framingham unrelated participants [32].

## Discussion

In collaboration with NCBI we have web-posted our unfiltered biomarker-genotype associations and linkage results to provide a resource to investigators seeking to understand and replicate their biomarker-genotype associations. We submit that the findings of highest priority for follow-up are associations that were detected by several statistical approaches. MCP1 was associated with 2 SNPs on chromosome 1 (rs4128725 and rs2494250) with p-values in the 10^-8 ^by FBAT, ≤ 10^-12 ^by GEE. Acknowledging that linkage is less powerful and accurate, we note that the 1.5 support interval for the MCP1 linkage peak (Winsorized maximum LOD 4.38) on chromosome 1 supports the GEE and FBAT analyses. Findings for CRP (chromosome 1), brain natriuretic peptide (chromosome 1) and Vitamin K % undercarboxylated osteocalcin (Chromosome 7) are also of potential priority for follow-up. We acknowledge that the ultimate validation of our findings will require replication in other cohorts and functional studies.

A fundamental challenge of GWAS tests is sorting through associations and prioritizing SNPs for follow-up. In the absence of external replication, one approach to synthesizing findings is to examine associations across similar biological domains, which may capture pleiotropy. We presented the exploratory analyses in Tables 3a and 3b, but reiterate that the findings will need to be examined in other cohorts.

### Do the findings represent true positive genetic associations?

It is notable that some of the associations with the strongest statistical support were for associations between a gene and its protein product (e.g. *CRP *gene and CRP concentration). Cis-acting regulatory variants have been shown to influence mRNA and protein levels for many genes [65]. Studies involving additional biomarker phenotypes and variants (e.g. Affymetrix 500 K Chip) should clarify whether cis- or trans-acting regulatory variants explain the greatest proportion of phenotypic variation.

With GWAS, which typically test for the association of 1000s of SNPs with multiple traits, it is difficult for any specific association to achieve genome wide significance. For instance, a strict Bonferroni correction for the 30 traits tested in the present study with both age/sex- and multivariable-adjusted models and 2 statistical methods (0.05/(70,987*30*2*2) would require a p = 5.9 × 10^-9^. We submit that the most significant association in the selected biomarker group, the *FCER1A *rs2494250 SNP with MCP1 concentrations achieved genome-wide significance with a GEE p = 1.0*10^-14 ^and a FBAT p = 3.5*10^-8^. It should be noted that rs2494250 and rs4128725 are in modest linkage disequilibrium (D' = 0.724 and r squared = 0.196) and hence, may be serving as proxies for the same causal SNP.

Several human and experimental studies suggest that the association between *FCER1A *and MCP1 concentrations is biologically plausible. *FCER1A *codes for the high affinity Fc receptor fragment for IgE. In vitro experiments with rat mast cells demonstrated that if aggregated the high affinity receptor for IgE (FcεRI) increased gene transcription and secretion of MCP1 [66]. Similarly, in mice mast cells if the FcεRI was occupied by small amounts IgE/antigen, MCP1 mRNA increased significantly [67]. In humans IgE and MCP1 concentrations are both increased in occupational asthma [68,69]. Similar to the animal data, human mast cells exposed to anti-IgE antibody or to IgE released MCP1 [70-72].

### Comparison with prior literature

Our efforts to compare our findings with associations previously reported in the literature underscore some of the challenges in genetic association studies. The *ICAM1 *gene did not have any markers within 60 kb on the Affymetrix 100K chip. Of the 4 genes that did have SNPs in the marker genomic region coding, only the *CRP *association was replicated in our cohort; however as noted above we [32], as well as others [20], have previously reported this association. For bilirubin concentrations we previously reported significant linkage to chromosome 2q telomere [39] and a significant association to a TA repeat in *UGT1A1*, under this linkage peak [40] in Framingham unrelated participants. However, there was no association between bilirubin concentrations and the 3 SNP within 60 kb of *UGT1A1*. The previously reported interleukin-6-*IL6 *and the MCP1-*CCL2 *associations were not replicated. Of note, our group previously reported that rs1024611 [in *CCL2*] was associated with MCP1 concentrations in unrelated participants [63]; the association was nowhere close to significant in the present report (FBAT p = 0.78; GEE p = 0.35) Possible explanations of the failure to confirm the previously reported Framingham study MCP1-*CCL2 *association may stem from the current report having a smaller sample size (n = 989), using different genetic markers, and being conducted with an additive genetic model in related participants, as opposed to the prior study using unrelated participants (n = 1602) with recessive and dominant models. In a recent meta-analysis of phenotype-genotype association studies, only about one third (8 of 25) of the associations examined were replicated [73]. There are many plausible explanations why we did not replicate previously reported phenotype-genotype associations. Previous reports could represent false positive findings, or the present and prior study cohorts may differ on key factors, which may modify the phenotype-genotype associations, or our lack of replication may represent a false negative report because of inadequate statistical power [73,74].

### Strengths and limitations

The strengths of the present study include a comprehensively characterized community-based cohort, with biomarker phenotypes routinely assessed with careful attention to quality control. However, the cohort was largely middle-aged to elderly, and white of European descent, so the findings may not be generalizable to individuals who are younger or of other ethnicity/racial descent. DNA was collected at the 5^th ^and 6^th ^examinations, which may have introduced a survival bias. In addition, our study was susceptible to false negative findings because of the moderate size of the cohort; we lacked power to detect modest associations. Conversely, similar to most GWAS, the reported associations and linkage may represent false positive findings from multiple statistical testing.

## Conclusions and future directions

The Framingham GWAS and the web posting of the unfiltered results represent a unique resource to discover potentially novel genetic influences on systemic biomarker variability. We acknowledge that the newly described associations will need to be replicated in other studies.

## Abbreviations

bp, base pair; Chr, chromosome;CRP, C-reactive protein; FBAT, Family Based Association Tests; GEE, Generalized Estimating Equations; GWAS, Genome-wide association studies; LD, linkage disequilibrium; LOD, logarithm of the odds (base 10); MCP-1, monocyte chemoattractant protein-1; SNPs, single nucleotide polymorphisms.

## Competing interests

The authors declare that they have no competing interests.

## Authors' contributions

EJB conceived of the FHS inflammation project, secured funding, planned the analyses, drafted and critically revised the manuscript. JD assisted in planning and conducting the analyses, and in writing and critically revising the manuscript. MGL planned the FHS inflammation project including assisting in securing funding, and planned and conducted analyses. KLL assisted in planning and conducting the analyses. SLB measured the vitamin data, assisted in planning the analyses and critically revising the manuscript. DRG participated in the study design and reviewed the manuscript. SK contributed to analyses of C-reactive protein and osteoprotegerin, and reviewed the manuscript. JFK assisted in securing the funding, supervised and organized the performance of the assays and reviewed the manuscript. MJK contributed to collecting the data base and revising the manuscript. JPL provided insights into the liver function test analyses and reviewed and approved the manuscript. JBM secured funding for and oversaw measurement of high-sensitivity TNFα concentrations and reviewed and approved the manuscript. SJR contributed to acquisition of the inflammation data, reviewing, revising and giving final approval to the manuscript. JR provided critical assistance in organizing the inflammatory marker data set, conducted quality control analyses and reviewed and gave final approval to the manuscript. RS was involved in revising the manuscript critically for important intellectual content and gave final approval of the version to be published. JAV assisted in securing funding for the inflammation project and revising the manuscript. TJW contributed to the analysis and interpretation of the data, and revision of the manuscript for important intellectual content. PWFW contributed to data acquisition, revision of the manuscript and final approval of the version submitted. PAW participated in 100K study design and reviewed and approved the manuscript. RSV provided critical input in conceiving the project, securing the funding, planning the analyses and critically revising the manuscript.
